# Update Vorhofflimmern: Die ESC-Leitlinien 2020 sowie aktuelle Daten zur frühen antiarrhythmischen Therapie

**DOI:** 10.1007/s00399-021-00749-4

**Published:** 2021-03-30

**Authors:** Ruben Schleberger, Andreas Rillig, Paulus Kirchhof, Andreas Metzner, Bruno Reissmann

**Affiliations:** Klinik und Poliklinik für Kardiologie, Universitäres Herz- und Gefäßzentrum UKE Hamburg, Universitätsklinikum Hamburg-Eppendorf, Martinistr. 52, 20246 Hamburg, Deutschland

**Keywords:** Atriale Tachyarrhythmien, Therapiealgorithmus, Medikamentöse antiarrhythmische Therapie, Katheterablation, EAST-AFNET 4, Atrial tachyarrhythmias, Therapy algorithm, Antiarrhythmic drug treatment, Catheter ablation, EAST-AFNET 4

## Abstract

Vorhofflimmern geht mit einer beträchtlichen Belastung für Patienten und das Gesundheitssystem einher. Jeder dritte heute 55-Jährige wird zeitlebens an Vorhofflimmern erkranken. Trotz Verbesserungen des Managements von Vorhofflimmern verbleibt ein erhöhtes Risiko für kardiovaskuläre Ereignisse. Die neuen Vorhofflimmer-Leitlinien der European Society of Cardiology stellen ein integratives Therapiekonzept in den Mittelpunkt. Der neue Therapie- und Diagnosealgorithmus „CC to ABC“ umfasst sowohl Diagnosestellung („confirm“) und Klassifizierung („characterise“) als auch Therapie („avoid stroke“, „better symptom control“, „comorbidities“). Neue orale Antikoagulanzien stehen unter Anwendung des CHA_2_DS_2_-VASC-Scores im Zentrum der Vorbeugung von Schlaganfällen. Neben der Frequenzregulierung werden rhythmuserhaltende Maßnahmen wie die medikamentöse antiarrhythmische Therapie oder die Katheterablation mit prognostischer Relevanz für bestimmten Patientengruppen empfohlen. Die Adressierung von Risikofaktoren und Komorbiditäten wie arterielle Hypertonie, Diabetes mellitus, Adipositas und Schlafapnoe wirkt ergänzend und sollte Teil jedes Behandlungskonzepts sein. Die im August 2020 publizierte EAST-AFNET 4-Studie zeigt als erste große randomisierte Studie, dass die frühe rhythmuserhaltende Therapie zusätzlich zur leitlinienbasierten Vorhofflimmertherapie zur Vermeidung von kardiovaskulärem Tod und Schlaganfällen beiträgt. In Anbetracht der Sicherheit von Antiarrhythmika und Katheterablation sollte die frühe Einleitung einer rhythmuserhaltenden Therapie bei allen Patienten in den ersten Monaten nach der Erstdiagnose von Vorhofflimmern erwogen werden, um positive Effekte nicht zu verpassen.

## Abstract (en)

Atrial fibrillation (AF) can be a significant burden for patients as well as the health care system. Every third 55-year-old will develop AF. Despite improvements of disease management, a significant risk for cardiovascular events remains. The current AF guidelines of the European Society of Cardiology focus on an integrative therapy approach. The new algorithm “CC to ABC” comprises recommendations for diagnosis (“confirm” and “characterise”) and treatment (“avoid stroke”, “better symptom control”, “comorbidities”) of AF. Direct oral anticoagulants administered according to the CHA_2_DS_2_-VASc score remain the corner stones of stroke prevention. Besides the concept of heart rate control, rhythm control therapy like antiarrhythmic drugs or catheter ablation is recommended to relieve symptoms and in certain patient groups for the improvement of prognosis. Therapy of comorbidities and reduction of risk factors like hypertension, diabetes mellitus, obesity and obstructive sleep apnoea should be part of any comprehensive treatment approach. The results of the randomized, prospective EAST-AFNET 4 trial were published in August 2020. The trial shows that an early rhythm control therapy can lead to a reduction of cardiovascular mortality and incidence of stroke additionally to guideline-based AF management. Given the safety profile and potential positive effects of antiarrhythmic drugs and catheter ablation, early initiation of rhythm control therapy should be considered in every patient during the first months after diagnosis of AF.

Vorhofflimmern geht mit einer beträchtlichen Belastung für Patienten und das Gesundheitssystem einher. Schätzungen zufolge wird zeitlebens jeder dritte heute 55-Jährige an Vorhofflimmern erkranken [1]. Vorhofflimmern ist mit gefährlichen kardiovaskulären Komplikationen assoziiert, insbesondere Herzinsuffizienz, Schlaganfall und kardiovaskulärem Tod [2]. Meilensteine der Behandlung von Vorhofflimmern, welche zur maßgeblichen Reduktion der Mortalität als auch Morbidität geführt haben, sind die orale Antikoagulation zur Verhinderung von Schlaganfällen sowie die Erkennung und Behandlung kardiovaskulärer Begleiterkrankungen. Zudem wird üblicherweise eine frequenzregulierende Therapie eingeleitet [3]. Dennoch treten auch unter genannten Maßnahmen weiterhin bei ca. 5 % der Patienten pro Jahr kardiovaskuläre Komplikationen auf [4, 5].

## Die aktuellen ESC-Leitlinien

**Figure 1:**
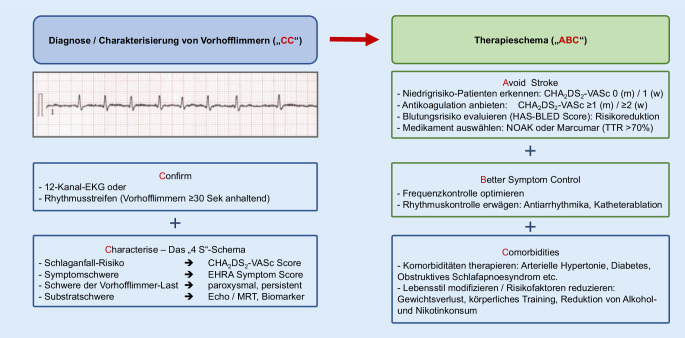

Die aktuellen Vorhofflimmer-Leitlinien der European Society of Cardiology (ESC) sind im August 2020 im Rahmen des ESC Online-Kongresses präsentiert worden [6]. Der Behandlungspfad „CC to ABC“ (CC: Confirm and characterise, ABC: Avoid stroke/anticoagulation; better symptom control; cardiovascular risk factors and concomitant diseases) zieht sich als roter Faden durch die Leitlinien, umfasst wesentliche Aspekte der Therapie des Vorhofflimmerns und kann Behandelnden als wichtiges Instrument dienen (Abb. 1).

### Screening

**Figure 2:**
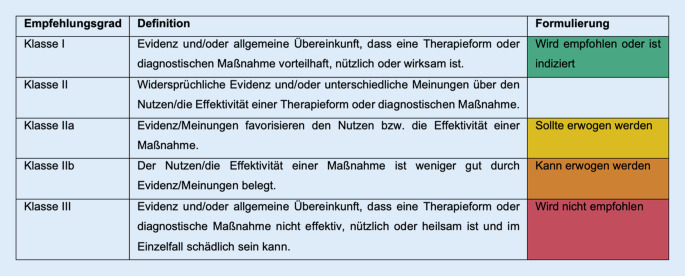

Die Notwendigkeit der frühen Diagnose des Vorhofflimmerns wird durch die hohe Zahl asymptomatischer Patienten sowie den relativ hohen Anteil ischämischer Schlaganfälle, die auf Vorhofflimmern zurückzuführen sind, begründet. Grundsätzlich empfehlen die Leitlinien wie auch in den Vorjahren ein kosteneffizientes, opportunistisches Screening bei allen Patienten > 65 Jahre (IB; Empfehlungs- und Evidenzgrade siehe Abb. 2). Strukturierte Empfehlungen bezüglich des weiteren Managements sollten vorgehalten werden (IB). Aufgewertet wurde die Empfehlung, für Patienten mit höherem Risikoprofil, wie z. B. Alter > 75 Jahre, ein systematisches Screening zu erwägen (IIaB).

### „CC – confirm and characterise“

Zur Diagnosestellung von Vorhofflimmern wird in den neuen Leitlinien ein 12-Kanal-EKG oder 1-/3-Kanal-EKG mit über 30 Sekunden anhaltendem Vorhofflimmern gefordert (IB).

Der neue Klassifizierungsalgorithmus „4S-AF Schema“ soll die wichtigsten Aspekte des Vorhofflimmerns nach Diagnosestellung strukturiert analysieren (IIaC). Die vier „S“ stehen für „stroke risk, symptom severity, severity of AF burden“ und „substrate severity“. Unter den Schlagworten verbergen sich die bekannten klinischen Tools CHA_2_DS_2_-VASc-Score, EHRA-Symptom-Score sowie das zeitliche Muster der Vorhofflimmerepisoden (paroxysmal, persistierend, langanhaltend persistierend und permanent). Die Evaluation der „substrate severity“ umfasst die Nutzung diagnostischer Mittel (Echokardiographie/MRT/Biomarker) und Erfassung der Komorbiditäten.

### Therapie von Vorhofflimmern

Im Zentrum der Leitlinien 2020 steht ein integratives Behandlungskonzept im Sinne einer interdisziplinären Betreuung durch Hausärztin/Hausarzt, Kardiologin/Kardiologe, Klinikteam, Apotheker/in u. a. (IIaB).

#### „A – anticoagulation/avoid stroke“

**Figure 3:**
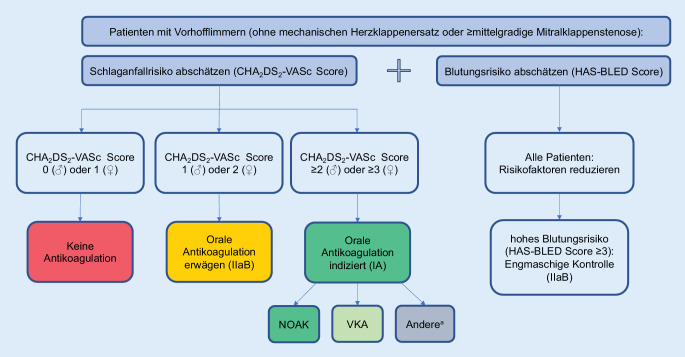

Der CHADS_2_DS_2_-VASc-Score als Kompromiss aus Praktikabilität und Genauigkeit ist maßgebend (IA; Abb. 3; Tab. 1). Das weibliche Geschlecht wird weiter im Score erfasst, da es sich um einen altersabhängigen Risikomodifikator handelt. Bei Vorliegen eines Risikofaktors für thrombembolische Ereignisse (ohne weibliches Geschlecht) sollte der Beginn einer oralen Antikoagulation erwogen werden (IIaB). Bei Patienten mit CHADS_2_DS_2_-VASc-Score 2 (Männer) bzw. 3 (Frauen) sollte eine orale Antikoagulation definitiv erfolgen (IA). Bei initial niedrigem Schlaganfallrisiko ist nach vier bis sechs Monaten eine Reevaluation zu empfehlen, da Risikofaktoren häufig erst nach Erstdiagnose von Vorhofflimmern zu Tage treten (IIaB).

**Table 1:** 

CHA_2_DS_2_-VASc-Score Risikofaktoren	Punkte
Herzinsuffizienz Zeichen/Symptome der Herzinsuffizienz oder objektive Hinweise auf eine reduzierte linksventrikuläre Ejektionsfraktion	+1
Hypertension Blutdruck in Ruhe > 140/90 mm Hg in 2 Messungen oder antihypertensive Therapie	+1
Alter ≥ 75 Jahre	+2
Diabetes mellitus Nüchtern-Glukose > 125 mg/dl (7 mmol/l) oder Behandlung mit Antidiabetika/Insulin	+1
Früherer Schlaganfall, TIA, Thrombembolie	+2
Vaskuläre Erkrankung Z. n. Myokardinfarkt, PAVK oder Verkalkung der Aorta	+1
Alter 65–74 Jahre	+1
Geschlecht (weiblich)	+1

*PAVK* periphere arterielle Verschlusskrankheit, *TIA* transitorische ischämische Attacke

Mit Beginn der oralen Antikoagulation sollte bei jedem Patienten auch das Blutungsrisiko evaluiert werden (IB). Bei hohem Blutungsrisiko (HAS-BLED-Score > 2) werden anstatt prophylaktischem Verzicht auf eine indizierte orale Antikoagulation eine Adressierung modifizierbarer Risikofaktoren und eine engmaschige klinische Überwachung empfohlen.

Bei Auswahl der Medikation zur oralen Antikoagulation zählen die neuen oralen Antikoagulanzien (NOAKs) wie gehabt aufgrund ihrer Effektivität, ihres Sicherheitsprofils sowie der einfachen Anwendung als Medikamente der ersten Wahl (IA). Im Fall einer Therapie mit Vitamin-K-Antagonisten sollte eine „time in therapeutic range“ > 70 % bei Ziel INR („international normalized ratio“) von 2,0–3,0 angestrebt werden (IB). Eine Dosisreduktion des jeweiligen NOAK abseits der evidenzbasierten Empfehlungen sollte vermieden werden.

#### „B – better symptom control“

Die beiden Hauptkonzepte der Vorhofflimmertherapie bleiben weiterhin die frequenzregulierende und die rhythmuserhaltende Therapie. Erstere kann hierbei als Basistherapie betrachtet werden. Keine Änderung ergibt sich beim anzustrebenden Frequenzziel mit < 110 Schlägen/min (IIaB). Die Empfehlung beruht u. a. auf Daten der RACE-, RACE II- sowie AFFIRM-Studie, wobei der Zielwert kontrovers diskutiert wird [7–9].

Die langfristige rhythmuserhaltende Therapie wird von den Autoren als symptomorientierte Therapie eingestuft und ist in der Regel Patienten mit symptomatischem Vorhofflimmern vorbehalten. Zur initialen Entscheidungsfindung bezüglich der Art der antiarrhythmischen Therapie (medikamentös vs. interventionell) steht in den Leitlinien 2020 der Patientenwunsch im Vordergrund. Die Katheterablation ist grundsätzlich sicher und der medikamentösen antiarrhythmischen Therapie bezüglich Symptomkontrolle und Erhalt des Sinusrhythmus überlegen [10–12].

Als Erstlinientherapie ist die Katheterablation bei Patienten mit paroxysmalem Vorhofflimmern eine IIaB-Empfehlung. Neu ist eine IIbC-Empfehlung der Erstlinientherapie für Patienten mit persistierendem Vorhofflimmern ohne schwerwiegende Risikofaktoren für Rezidive.

Flecainid und Propafenon sollten nur bei Patienten ohne strukturelle Herzerkrankung eingesetzt werden (IA). Amiodaron ist das Medikament mit der höchsten Effektivität und kann auch bei Patienten mit struktureller Herzerkrankung und akuter Herzinsuffizienz eingesetzt werden [13]. Engmaschige klinische sowie laborchemische Überwachungen sind aufgrund der häufigen Nebenwirkungen der Amiodarontherapie notwendig, sodass wenn möglich anderen Antiarrhythmika der Vorzug gegeben werden sollte (IA). Dronedaron hat ein besseres Sicherheitsprofil als Amiodaron, jedoch auch eine niedrigere Effektivität [14, 15]. Der Einsatz wird als Langzeittherapie bei Patienten mit erhaltener oder leicht reduzierter (stabiler) linksventrikulärer Ejektionsfraktion empfohlen (IA). Im Unterschied zu einer Therapie mit Flecainid oder Propafenon schließt dies Patienten mit koronarer und hypertensiver Herzerkrankung ein. Bei allen Antiarrhythmika werden EKG-Kontrollen zu Beginn der Therapie sowie nach ein bis zwei Wochen (Flecainid/Propafenon) bzw. vier Wochen (Amiodaron/Dronedaron) empfohlen.

Für ausgewählte Patientenkollektive, wie z. B. Patienten mit eingeschränkter linksventrikulärer Ejektionsfraktion, konnte eine Verbesserung der Mortalität durch die Katheterablation gezeigt werden (CASTLE-AF-Studie; [16]). Auf Grundlage dieser aktuellen Daten wird eine Klasse-IIaB-Empfehlung für die symptomunabhängige Katheterablation bei Patienten mit Herzinsuffizienz und reduzierter linksventrikulärer Ejektionsfraktion abgegeben. Weiterhin gibt es nun eine Empfehlung IB der Katheterablation für Patienten mit hochgradigem Verdacht auf Tachymyopathie.

Als Zweitlinientherapie nach frustraner bzw. nichttolerierter medikamentöser antiarrhythmischer Therapie wird die Katheterablation sowohl für Patienten mit paroxysmalem als auch mit persistierendem Vorhofflimmern empfohlen (IA).

Die orale Antikoagulation sollte nach Katheterablation bei allen Patienten für mindestens zwei Monate und im Anschluss nach CHA_2_DS_2_-VASc-Score fortgeführt werden (IC).

#### „C – cardiovascular risk factors and concomitant diseases“

Das Auftreten von Vorhofflimmern wird u. a. durch den Lebensstil, kardiovaskuläre Risikofaktoren und kardiale Grunderkrankungen beeinflusst. Die RACE III-Studie zeigte, dass die gezielte Behandlung von Risikofaktoren und Grunderkrankungen bei Patienten mit Herzinsuffizienz zu einer Stabilisierung des Sinusrhythmus führt [17]; diese wird daher als Teil der Behandlung des Vorhofflimmerns empfohlen (IB).

## Frühe antiarrhythmische Therapie – aktuelle Studiendaten

Die Ergebnisse der ebenfalls im Rahmen des ESC-Kongresses 2020 vorgestellten EAST-AFNET 4-Studie („early treatment of atrial fibrillation for stroke prevention trial“; Tab. 2) fordern möglicherweise schon ein erstes fokussiertes Update der Leitlinie [4].

**Table 2:** 

Studie (Jahr)	Studienpopulation	Behandlungsgruppen	Endpunkte	Ergebnisse
EAST-AFNET 4 (2020) [4]	Patienten mit kürzlich diagnostiziertem Vorhofflimmern (Erstdiagnose ≤ 1 Jahr vor Studieneinschluss; *n* = 2789) Risikofaktoren für thrombembolische Ereignisse (Alter > 75 Jahre, Z. n. TIA/Schlaganfall oder ≥ 2 der folgenden Kriterien: Alter > 65 Jahre, weibliches Geschlecht, Herzinsuffizienz, arterieller Hypertonus, Diabetes mellitus, schwere KHK, chronische Niereninsuffizienz (Stadium III/IV), LV-Septumdicke > 15 mm)	*Frühe rhythmuserhaltende Therapie*: PVI und/oder AAD. EKV nach Notwendigkeit *Kontrollgruppe*: Frequenzkontrolle, rhythmuserhaltende Therapie bei persistierenden Symptomen Fortführung von OAK nach CHA_2_DS_2_-VASc-Score in beiden Gruppen	Primärer Endpunkt: kardiovaskulärer Tod, Schlaganfall, Hospitalisierung aufgrund von Herzinsuffizienz oder akutem Koronarsyndrom Primärer Sicherheitsendpunkt: Tod, Schlaganfall, Auswahl von SAEs Mediane Dauer des Follow-ups: 5,1 Jahre	249 (3,9 pro 100 Personenjahre) Ereignisse des primären Endpunkts (rhythmuserhaltende Therapie) vs. 316 (5,0 pro 100 Personenjahre) Ereignisse (Kontrollgruppe); HR 0,79; 96 % KI [0,66–0,94]; *p* = 0,005 Primärer Sicherheitsendpunkt: 231 (16,6 %) Patienten mit rhythmuserhaltender Therapie vs. 223 (16,0 %) Patienten der Kontrollgruppe; *p* < 0,001
EARLY-AF (2021) [12]	Patienten mit kürzlich diagnostiziertem (Erstdiagnose ≤ 2 Jahre), therapienaivem (keine regelhafte AAD-Einnahme), symptomatischem, paroxysmalem Vorhofflimmern (*n* = 303)	*Interventionsgruppe*: PVI mittels Kryoballon *Kontrollgruppe*: AAD nach Leitlinie (Auftitration während 90 Tage Blanking-Periode) Implantation eines Ereignisrekorders in beiden Gruppen	Primärer Endpunkt: erstes Rezidiv einer atrialen Tachyarrhythmie (> 30 s) ab Ende der Blanking-Periode (90 Tage post interventionem) Sekundäre Endpunkte (Auswahl): symptomatische Rezidive; Vorhofflimmerlast; SAE Follow-up-Dauer: 12 Monate	Primärer Endpunkt: 66 (42,9 % der Patienten der Interventionsgruppe) vs. 101 (67,8 % der Patienten der Kontrollgruppe); HR 0,48; 95 % KI [0,35–0,66]; *p* < 0,001 Symptomatische Rezidive: 17 (11,0 %) vs. 39 (26,2 %); HR 0,39 [0,22–0,68] Vorhofflimmerlast (Median): 0 %; IQR (0–0,08) vs. 0,13 %; IQR (0–1,60) SAE: 5 (3,2 %) vs. 6 (4,0 %); RR 0,81; 95 % KI [0,25–2,59]
STOP-AF First (2021) [18]	Patienten mit therapienaivem (AAD < 7 Tage), symptomatischem, paroxysmalem Vorhofflimmern (*n* = 203)	*Interventionsgruppe*: PVI mittels Kryoballon *Kontrollgruppe*: AAD nach Leitlinie (Auftitration während 90 Tage Blanking-Periode)	Primärer Endpunkt: Behandlungserfolg, definiert als Freiheit von Rezidiv einer atrialen Tachyarrhythmie (> 30 s) ab Ende der Blanking-Periode (90 Tage post interventionem), linksatriale Reablation, frustrane primäre Ablation, Kardioversion, Nutzung von AAD nach Blanking-Periode (nur Interventionsgruppe) Primärer Sicherheitsendpunkt (nur Interventionsgruppe): Perikarderguss innerhalb 30 Tagen post interventionem, Pulmonalvenenstenose, ösophagoatriale Fistel, permanente Phrenikusparese, TIA/Schlaganfall, Myokardinfarkt, schwere Gefäßkomplikation, schwere Blutung (innerhalb von 7 Tagen postprozedural) Follow-up-Dauer: 12 Monate	Primärer Endpunkt (Behandlungserfolg; Kaplan-Meier-Schätzer): 74,6 %; 95 % KI [65,0–82,0] (Interventionsgruppe) vs. 45,0 %; 95 % KI [34,6–54,7] (Kontrollgruppe); *p* < 0,001 Primärer Sicherheitsendpunkt (Kaplan-Meier-Schätzer): 1,9 %; 95 % KI [0,5–7,5] SAE: 15 (14 %) vs. 14 (14 %); ns

*AAD* antiarrhythmische medikamentöse Therapie, *EKV* Kardioversion, *LV* linker Ventrikel, *HR* Hazard-Ratio, *IQR* „interquartile range“, *KI* Konfidenzintervall, *KHK* koronare Herzerkrankung, *PVI* Pulmonalvenenisolation, *RR* Risikoreduktion, *SAE* „serious adverse event“, *TIA* transitorische ischämische Attacke

Die Studie prüfte die Hypothese, ob eine früh begonnene rhythmuserhaltende Therapie das Auftreten von kardiovaskulären Komorbiditäten und kardiovaskulären Ereignissen verhindern kann. Insgesamt 2789 Patienten mit Vorhofflimmern wurden im Median 36 Tage nach Diagnosestellung in 135 Zentren randomisiert. In der Interventionsgruppe erfolgte eine frühe medikamentöse oder interventionelle rhythmuserhaltende Therapie. In der Kontrollgruppe erfolgte die Therapie nach den gültigen Leitlinien, d. h. zunächst Frequenzregulierung und Hinzufügen einer rhythmusstabilisierenden Therapie bei weiterbestehenden Symptomen. Das mediane Follow-up betrug 5,1 Jahre. Ein Ereignis des zusammengesetzten primären Endpunkts (kardiovaskulärer Tod, Schlaganfall, Hospitalisierung mit Herzinsuffizienz oder akutem Koronarsyndrom) trat bei 249 Patienten mit früher rhythmuserhaltender Therapie und bei 316 Patienten mit konventioneller Behandlung auf (Hazard-Ratio 0,79; 96 % Konfidenzintervall [0,66–0,94]; *p* = 0,005). Die Einzelkomponenten des kombinierten Endpunkts traten in der Gruppe mit früher rhythmuserhaltender Therapie ebenfalls vermindert auf. Die Gesamtmortalität war statistisch nicht unterschiedlich (9,9 % bei Patienten mit früher rhythmuserhaltender Therapie, 11,8 % bei Patienten der Kontrollgruppe). „Serious adverse events related to rhythm control“ waren insgesamt sehr selten. So wiesen nur 28/1395 Patienten mit früher rhythmuserhaltender Therapie (0,28 %/Jahr) und 9/1394 Patienten in der Kontrollgruppe (0,13 %/Jahr) schwerwiegende unerwünschte Ereignisse wegen antiarrhythmischer Therapie auf. Ablationskomplikationen fanden sich bei 9/1394 Patienten mit früher rhythmuserhaltender Behandlung (0,13 %/Jahr) und 2/1394 Patienten in „usual care“ (0,03 %/Jahr; *p* < 0,001).

Die EAST-AFNET 4-Studie zeigte als erste große randomisiert-kontrollierte Studie, dass die frühe rhythmuserhaltende Therapie zur Vermeidung von kardiovaskulärem Tod und Schlaganfällen wesentlich beiträgt. Für Patienten mit neu aufgetretenem Vorhofflimmern sollte auch im Hinblick auf die erst kürzlich erschienenen Leitlinien das Therapiekonzept hin zu einer frühen rhythmuserhaltenden Therapie angepasst werden.

Bestärkt wurden diese positiven Ergebnisse bereits Ende 2020 durch zwei nordamerikanische Studien, die ebenfalls die Relevanz der frühen Vorhofflimmertherapie untersuchten. Die EARLY-AF-Studie und die STOP-AF-First-Studie zeigten beide unabhängig voneinander die Überlegenheit der interventionellen rhythmuserhaltenden Therapie mittels Kryoballonablation gegenüber einer medikamentösen antiarrhythmischen Therapie als Initialtherapie bei Patienten mit paroxysmalem Vorhofflimmern bei vergleichbarer Sicherheit (Tab. 2; [12, 18]).

Als Schlussfolgerung sollte in Anbetracht der Sicherheit von Antiarrhythmika und Katheterablation sowie in Kenntnis der Wirkung auf kardiovaskuläre Komplikationen eine frühe Einleitung einer rhythmuserhaltenden Therapie bei allen Patienten in den ersten Monaten nach Erstdiagnose von Vorhofflimmern erwogen werden, um positive Effekte nicht zu verpassen. Dies ist eine wesentliche Veränderung, die in ein Update der Vorhofflimmer-Leitlinien einfließen sollte.
